# A Simple External Fixator Construct for Assessment of Intra-operative Hip-Knee-Ankle Axis Alignment in Lower Limb Deformity Correction Surgery

**DOI:** 10.5704/MOJ.2007.004

**Published:** 2020-07

**Authors:** T Verma, VH Chavali

**Affiliations:** Department of Orthopaedics, Medical College Baroda and Associated Sir Sayajirao General (SSG) Hospital, Vadodara, India

Coronal plane deformities of lower limb are often challenging and require a thorough pre-operative planning, accurate intra-operative assessment and execution. Intra-operative confirmation of correct restoration of lower limb alignment [Hip-Knee-Ankle (HKA) axis] is more often than not assessed by gross visual inspection, which can be fallacious. Various methods described in literature for intra-operative assessment include cable method, axis board, specialised alignment rods (used in total knee arthroplasty) with a connector, computer assisted surgeries, etc^1, 2, 3^. Novel technologies like navigation and robotic surgery are very costly and not easily available in all centres, especially in developing countries. We describe a simple method to assess the HKA alignment while performing coronal deformity correction around the knee using external fixator components which are commonly available with every centre.

**Fig. 1:**
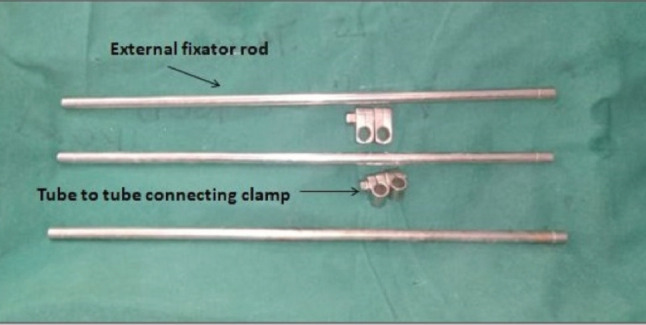
Apparatus required for alignment guide.

**Fig. 2:**
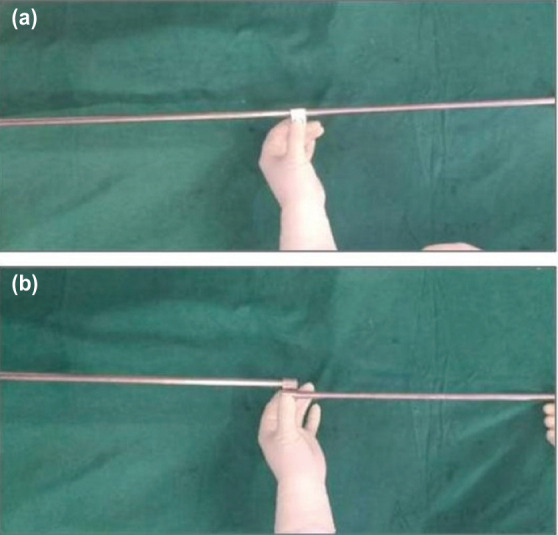
Method of assembling the external fixator construct. (a) Profile in which rods are aligned in exact straight line in one plane. (b) Profile in another plane (90° to previous one) in which connected rods are parallel, but not along same straight line.

**Fig. 3:**
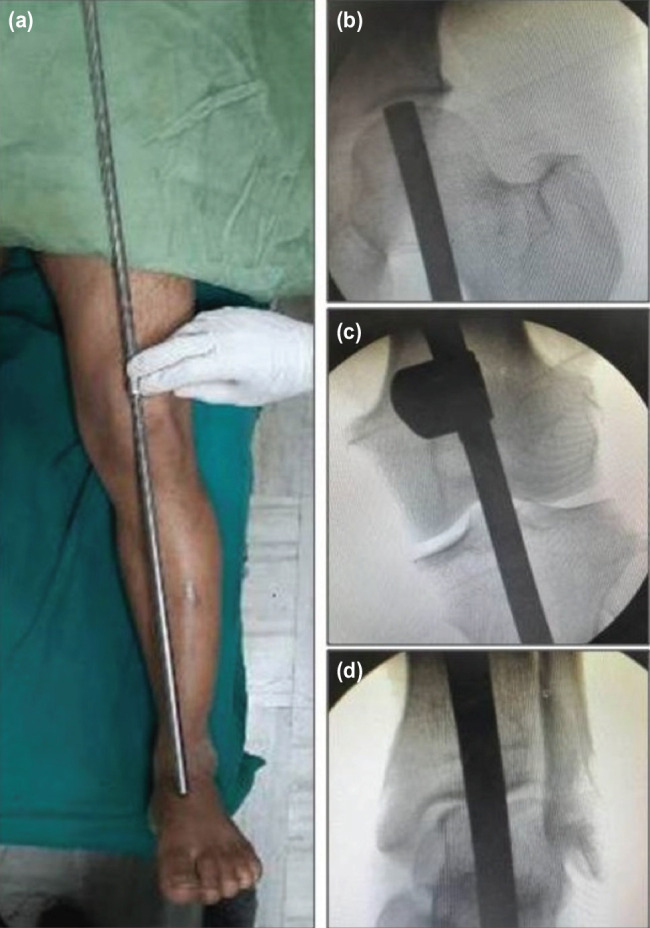
Demonstration of use of the external fixator construct as an alignment guide. (a) Alignment guide construct placed along left lower limb. (b) Upper end placed along the femoral head centre under C-arm guidance. (c) Alignment assessed at knee with C-arm image. (d) Lower end placed along centre of ankle joint.

Apparatus required for assessment of alignment includes external fixator rods and tube to tube connector clamps (Fig. 1). Connect two or three external fixator rods using tube to tube connector clamp (as shown in Fig. 2) to match the length required. By doing this, the rods will be aligned in exact straight line in one plane (Fig. 2a), while in another plane (90° to previous one), they will be in different parallel lines (Fig. 2b). This construct can then be used as an alignment guide by keeping it in the profile where rods are in straight line. Under C-arm guidance, one can place the upper end and lower end of the construct along the centre of femoral head and the centre of ankle joint, respectively and assess where exactly is it passing through the knee joint (Fig.3).

We have shown the application of this method using external fixator set from Greens® Surgicals Private Limited. However, external fixators from any manufacturer can be utilised for this purpose. We believe this simple method can be easily used by any orthopaedic surgeon in any centre without any additional efforts or cost.
